# Temperature-Induced Phase Transition Characteristics of [001]-Oriented 0.93Pb(Zn_1/3_Nb_2/3_)O_3_-0.07PbTiO_3_ (PZN-7%PT) Single Crystal by Using Piezoresponse Force Microscopy

**DOI:** 10.3390/ma14040855

**Published:** 2021-02-10

**Authors:** Hongli Wang, Kaiyang Zeng

**Affiliations:** 1 The Key Lab of Guangdong for Modern Surface Engineering Technology, National Engineering Laboratory for Modern Materials Surface Engineering Technology, Institute of New Materials, Guangdong Academy of Sciences, Guangzhou 510650, China; wanghongli@gdinm.com; 2 Guangdong Provincial Key Laboratory of Advanced Energy Storage Materials, School of Materials Science and Engineering, South China University of Technology, Guangzhou 510640, China; 3 Department of Mechanical Engineering, National University of Singapore, 9 Engineering Drive 1, Singapore 117576, Singapore

**Keywords:** phase transition, temperature, PZN-7%PT, PFM

## Abstract

The evolution of the domain structures of [001]-oriented relaxor ferroelectric 0.93PbZn_1/3_Nb_2/3_O_3_-0.07PbTiO_3_ (PZN-7%PT) single crystals as a function of temperature was investigated in situ by using piezoresponse force microscopy (PFM). It was found that the local domain structure of PZN-7%PT single crystals at room temperature is rhombohedral with nanoscale twins. Temperature-dependent domain structures showed that the phase transition process is a collective process and that the sample underwent a sequence of rhombohedral (R) → monoclinic (Mc) → tetragonal (T) → cubic (C) phase transformations when the temperature increased from 25 °C to 170 °C. The results provide direct observation of the phase transition evolution of PZN-7%PT single crystals as a function of temperature, which is of great significance to fully understand the relationships between the domain structure and phase structure of PZN-7%PT single crystals.

## 1. Introduction

Relaxor-PbTiO_3_ (relaxor-PT) ferroelectric single crystals, such as (1-*x*)Pb(Mg_1/3_Nb_2/3_)O_3_-*x*PbTiO_3_ and (1-*x*)Pb(Zn_1/3_Nb_2/3_)O_3_-*x*PbTiO_3_, are promising materials for next-generation high-performance transducers, sensors, and actuators because of their desirable piezoelectric coefficients (*d*_33_ > 2000 pC N^−1^) and electromechanical coupling factors (*k*_33_ > 0.9) [1,2,3]. These extraordinary properties are closely related to the morphotropic phase boundary (MPB) between the rhombohedral (R) and tetragonal (T) phases in the phase diagrams of relaxor-PT single crystals after poling along the [001] direction [4]. It is generally accepted that the ferroelectric phase transition temperature and Curie temperature (*T*_C_) are crucial to the performance of ferroelectric materials because these temperatures not only affect the material structures but also determine the temperature usage range and thermal stability of the material properties [5,6,7,8,9,10]. Because a [001]-oriented PZN-7%PT single crystal is in the MPB region, it is of great significance to investigate the phase transition process for understanding the relationship between the phase structure and material properties.

Numerous studies have been conducted to understand the relationship between the temperature and the phases in relaxor-PT ferroelectrics. It was reported that PZN-x%PT single crystals with compositions on the left side of the MPB, such as PZN-4.5%PT and PZN-6%PT, went through an R → T → C (cubic phase) transition with increasing temperature [7,11,12,13,14]. PZN-7%PT (around the MPB) single crystals were reported to experience an R → (R + RNT + T + TNT) → (T + TNT) → C transformation sequence (TNT and RNT are short for tetragonal and rhombohedral nano-twin states, respectively) [15,16]. PZN–9%PT (around the MPB) underwent an (R + T) → R → T → C phase transition [17], and Chang et al. further demonstrated that PZN-9%PT underwent a sequence of R + T → T → C phase transformation upon heating [18]. For compositions on the right side of the MPB, PZN-10.5%PT was reported to undergo a T → C transformation [19]. As can be seen from the results of previous studies, no agreement has been reached on the phase transition of relaxor–PT single crystals with increasing temperature.

In the last several decades, the phase transition dynamics in relaxor–PT ferroelectrics have been investigated by means of neutron scattering [7,11,20,21,22,23,24], X-ray diffraction [16,19,21], Raman scattering [25,26], Brillouin scattering [27], and infrared reflectivity [28,29]. However, few of the above methods can directly observe the in situ evolution of domain structures with different phase structures on a micro- or nanometer scale in real time. Piezoresponse force microscopy (PFM) is a state-of-the-art technique for visualizing and characterizing domain-related features in ferroelectric materials on the nanometer scale [30], and the latest developments in this technique have allowed the in situ detection of domain structures with increasing temperatures.

In this work, the three-dimensional domain structure of PZN-7%PT with increasing temperature from room temperature (approximately 25 °C) to 170 °C was explored using the PFM technique. It was found that the phase transition sequence of the [001]-oriented PZN-7%PT single crystals with increasing temperature was R → Mc → T → C, which confirms the existence of the monoclinic (Mc) phase and clarifies the polarization rotation path of PZN-7%PT single crystals with increasing temperature. As far as we know, this is the first report to employ PFM to directly observe the temperature-dependent phase transition of relaxor–PT single crystals in real time.

## 2. Materials and Methods

A PZN-7%PT single crystal (Microfine Materials Technology Pte. Ltd., Singapore, Singapore) grown by an improved high-temperature flux growth technique with PbO-based fluxes was used in the experiment [31]. The crystal was oriented using the Laue back-reflection method and sliced into specimens of [100]^L^/[010]^W^/[001]^T^. The surfaces of the samples were first polished with SiC papers, followed by fine polishing with 0.3 μm and 0.05 μm alumina powder using a water-cooled semi-auto polisher (Struers LaboForce-3, Ballerup, Denmark). The final size of the samples was approximately 4 mm (L) × 4 mm (W) × 0.5 mm (T).

**Figure 1:**
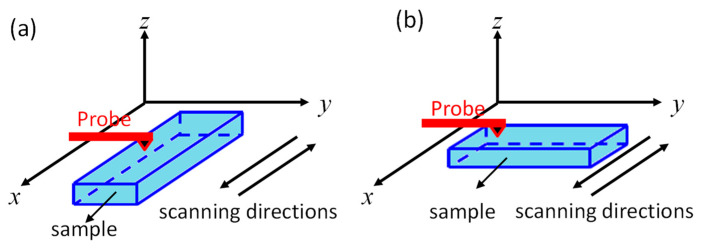
Schematic diagram of the sample orientation and scanning directions: (**a**) before 90° rotation and (**b**) after 90° rotation.

PFM measurements were performed using a model MFP-3D scanning probe microscopy system (Asylum Research, Santa Barbara, CA, USA). A conductive PtIr-coated silicon tip (PPP-NCSTPt, Nanosensors, Neuchâtel, Switzerland) with a length of 150 m, a resonant frequency of 160 kHz, and a spring constant of 7.4 nN/nm was used to image the domain structures. The PFM images were obtained with the dual-AC resonance tracking (DART) mode [32], in which two different drive frequencies were used to track the resonant frequency between the sample and the cantilever. The drive amplitude for PFM scanning was 1 V. Figure 1 shows the schematic diagram of the sample orientations and the scanning directions. The scanning directions of the probe were along the x-axis. Before 90° rotation, the length of the sample was along the x-axis, and so the PFM scanning was along the length direction of the sample. By scanning the sample in the lateral and vertical directions, PFM data along the length (x-LPFM) and thickness (VPFM) directions were obtained. Then, the sample was rotated 90°, with the width of the sample along the x-axis, so PFM data along the width direction (y-LPFM) were obtained. In this study, the definitions of x-LPFM, y-LPFM, and VPFM were based on the coordinate system of the sample.

## 3. Results and Discussion

**Figure 2:**
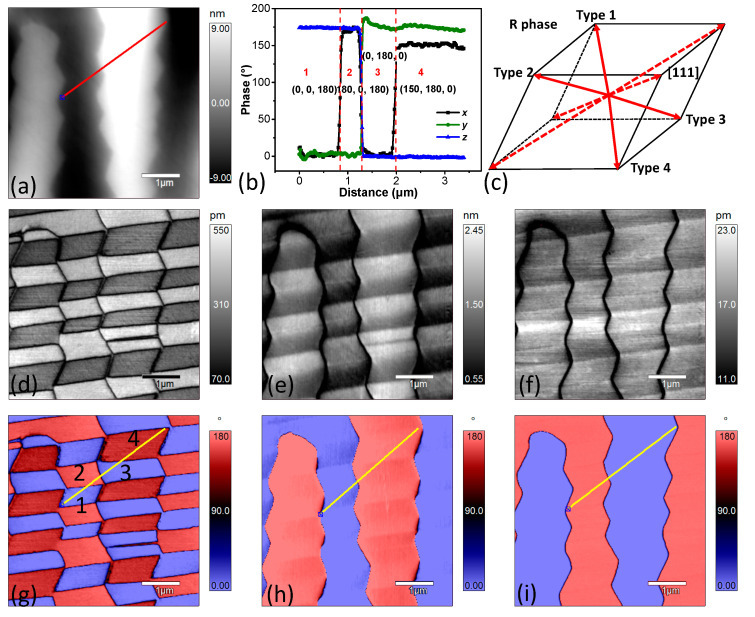
Topography and piezoresponse force microscopy (PFM) images of the PZN-7%PT single crystal at room temperature: (**a**) topography; (**d**–**f**) amplitude images of length (x-LPFM), width (y-LPFM), and thickness (VPFM), respectively; and (**g**–**i**) phase images of x-LPFM, y-LPFM, and VPFM, respectively. (**b**) extracted profiles of the phase data along the lines indicated in (**g**–**i**); and (**c**) possible polarization orientations in the rhombohedral phase.

**Figure 3:**
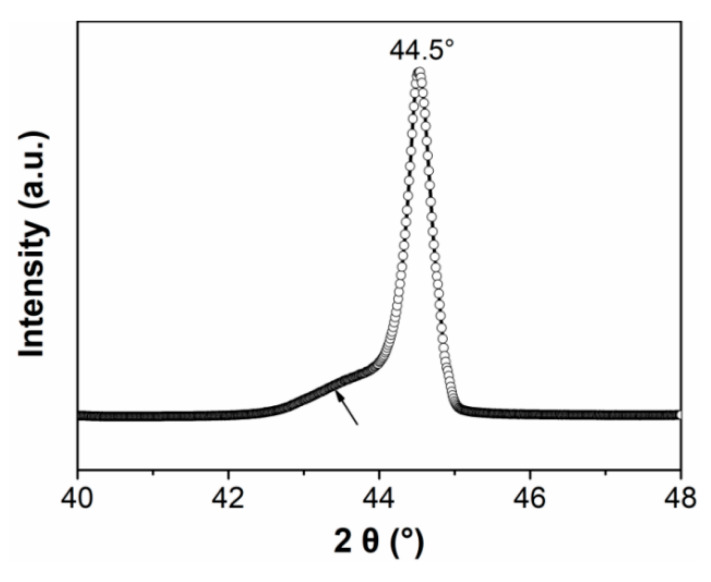
X-ray diffraction (XRD) profiles of the PZN-7%PT single crystal.

Figure 2 shows the topography, PFM amplitude, and phase images of the PZN-7%PT single crystal with a scan area of 5 × 5 μm. Both the y-LPFM (Figure 2e,h) and VPFM (Figure 2f,i) images show two types of contrast, and the contrasts agree with that of the topography (Figure 2a). However, the contrast of the x-LPFM (Figure 2d,g) images is not in accordance with that of the topography. Figure 2b shows the profile of the phase angle data across the lines in Figure 2g–i. There are four combinations of the PFM phase angle data in Figure 2b, indicating that there were four types of domain structures with different polarization orientations at room temperature. Figure 3 shows the X-ray diffraction patterns of the PZN-7%PT single crystal. The main peak at 2θ = 44.50° represents the rhombohedral phase [18], and the four possible polarization orientations of the rhombohedral phase, shown in Figure 2b, are depicted in Figure 2c. In addition, the low-intensity broader peak on the left side of the main peak can be interpreted as the effect of the polishing stress [33]. The phase angle difference of the x-LPFM in the type 3 and type 4 domains (as marked in Figure 2b,c) is approximately 150° rather than the typical 180°, which represents opposite polarization directions. This may be caused by polarization rotation, which could be induced by the stress generated in the surface layer during the grinding and polishing processes [33,34]. The alternatively distributed striped structure in the x-LPFM amplitude (Figure 2d) and phase images (Figure 2g) is an indication of the existence of twin structures. Therefore, the local phase structure of the PZN-7%PT single crystal at room temperature is rhombohedral with nanoscale twins.

**Figure 4:**
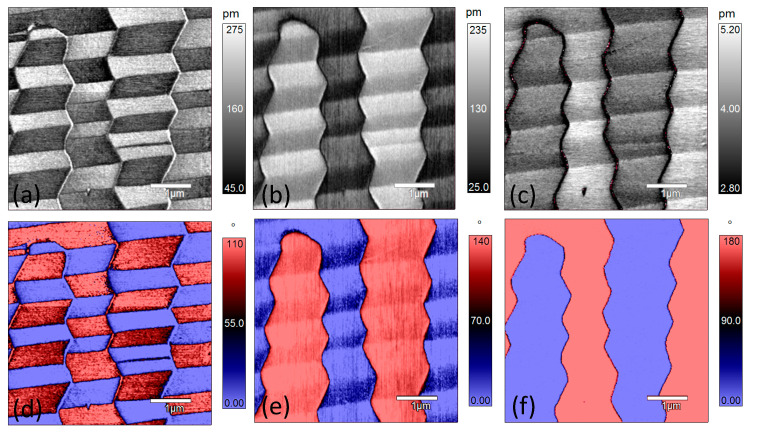
PFM amplitude and phase images of [001]-cut PZN-7%PT single crystal at 50 °C: (**a**–**c**) amplitude images of x-LPFM, y-LPFM, and VPFM, respectively; and (**d**–**f**) phase images of x-LPFM, y-LPFM, and VPFM, respectively as in Figure 2 at 75 °C. The width of the stripes in the x-LPFM amplitude (Figure 5a) and phase (Figure 5d) images become narrower than those at room temperature and 50 °C, and the number of domain walls increases. This is an indication of polarization rotation or phase transition with increasing temperature because the increase of domain wall area and the formation of sub-domains can help absorb the strain generated during the polarization rotation or phase transition process [35]. Additionally, the stripe-featured contrast in the y-LPFM amplitude image (Figure 5b) becomes ambiguous and the phase angle difference (Figure 5e) continues to decrease. Another change is that the y-LPFM and VPFM phase contrasts in the circled regions in Figure 5b,c,e,f became opposite to those at lower temperatures. This may be owing to the stability loss of the compensational surface charge at the top end of the domain at high temperatures [36,37]. The sample further was heated to 100 °C, and the PFM amplitude and phase did not show any significant change (Figure S1).

Figure 4 shows the PFM amplitude and phase images of the same region as in Figure 2 at 50 °C. Comparing the amplitude and phase images of x-LPFM at room temperature (Figure 2d) with those at 50 °C (Figure 4a), the general PFM phase contrast does not change much but the phase angle difference decreases from 180° at room temperature to approximately 110° at 50 °C. For the y-LPFM amplitude (Figure 4b) and phase images (Figure 4e), the general contrast remains the same as that at room temperature, but the phase angle difference decreases from 180° to approximately 140°. Figure 4c,f demonstrate the VPFM images, and both the amplitude and phase contrast (180°) remain the same as those at room temperature.

**Figure 6:**
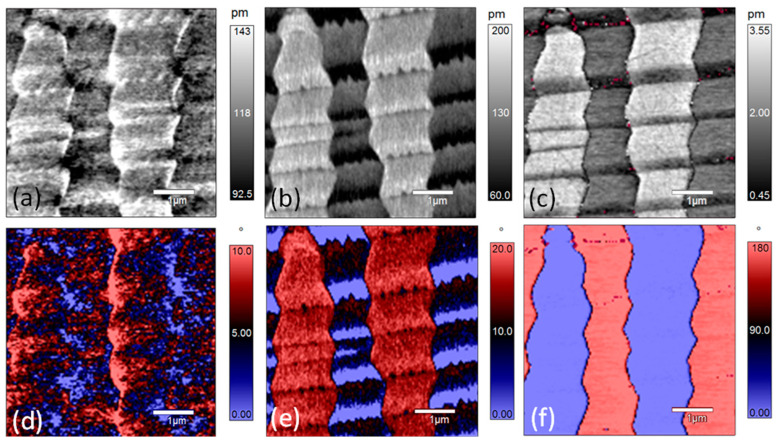
PFM amplitude and phase of [001]-cut PZN-7%PT single crystal at 120 °C: (**a**–**c**) amplitude images of x-LPFM, y-LPFM, and VPFM, respectively; and (**d**–**f**) phase images of x-LPFM, y-LPFM, and VPFM, respectively.

**Figure 7:**
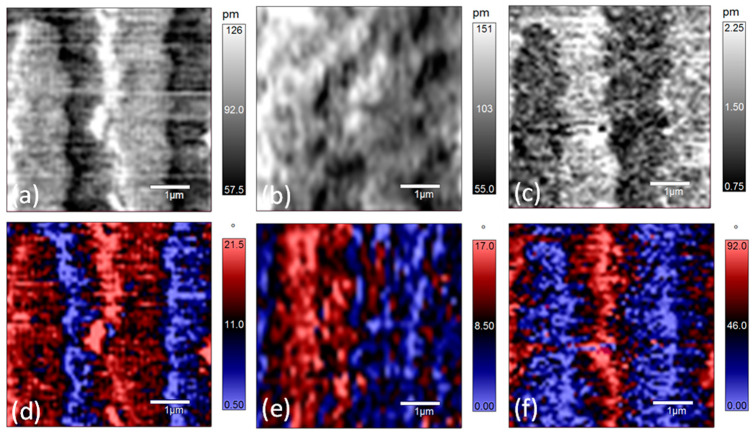
PFM amplitude and phase of [001]-cut PZN-7%PT single crystal at 170 °C: (**a**–**c**) amplitude images of x-LPFM, y-LPFM, and VPFM, respectively; and (**d**–**f**) phase images of x-LPFM, y-LPFM, and VPFM, respectively.

Figure 6 shows the PFM amplitude and phase images at 120 °C. The profiles of the x-LPFM amplitude and phase images become ambiguous and scattered. Comparing the contrasts of the y-LPFM amplitude and phase images at 120 °C with those at lower temperatures, the domain structure changed to show stripe-like features, especially for the domains with blue-colored regions (Figure 6e), which is an indication of the nano-twin structure. In addition, it can be seen that the profiles of the domain walls in the y-LPFM images become rougher. According to pervious reports, the motion of ferroelectric domain walls appears to be a collective process [34]. With the increase of the temperature, the arrangement of polarization within domains can be energetically favorable, but the strain generated during the polarization rotation process accumulates. In order to maintain the principle of energy minimization, the stress can be released by increasing the domain wall [38]. This is why the domain wall motion happens. Since the material is not perfect, and there are defects like acceptor-oxygen-vacancy defect pairs [39,40]. Therefore, the domain wall movement can be strongly inhibited, which is called domain wall pinning. This may be also the reason for domain wall roughening. When the temperature increases from 120 °C to 170 °C, the contrasts in the x-LPFM and y-LPFM images do not show any apparent change, but the phase difference in the VPFM image decreases gradually (Figures S2 and S3). Figure 7 shows the PFM amplitude and phase images of the PZN-7%PT single crystal at 170 °C. It can be seen that the domain boundaries are blurred, and nanosized polar clusters are visible.

**Figure 8:**
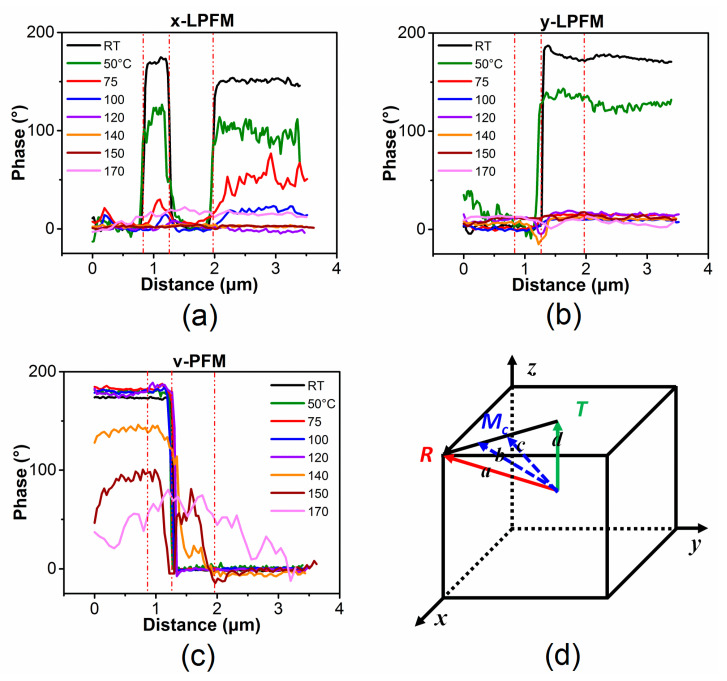
(**a**–**c**) Extracted profiles of the PFM phase in x-LPFM, y-LPFM, and VPFM along the line indicated in Figure 2a from room temperature to 170 °C; and (**d**) possible polarization rotation path (from R phase to T phase) of the [001]-cut PZN-7%PT single crystal.

Figure 8 summarizes the x-LPFM, y-LPFM, and VPFM phase data along the line marked in Figure 2a. At room temperature, there is an approximately 180° phase angle difference in the x-LPFM, y-LPFM, and VPFM images, and the XRD data reveal that the local domain structure of the PZN-7%PT single crystal is rhombohedral. When the temperature increases to 100 °C, the phase angle difference in the x-LPFM and y-LPFM decreases, whereas the phase angle difference in the VPFM remains 180°. This suggests that the local symmetry of the sample became tetragonal at 100 °C. The twinned domain structures shown in Figure 6b,e indicate that the sample had a tetragonal structure with nano-twins at 120 °C. The appearance of a twin structure can help accommodate the stress induced during the polarization rotation process [16,34]. Because the phase contrasts in the x-LPFM and y-LPFM decrease gradually from 25 °C to 100 °C, the possible R → T polarization rotation path may be via a monoclinic phase (Mc), as shown in the Figure 8d, which agrees with the results of previous studies [41,42,43,44]. When the temperature further increases to 140 °C, the VPFM phase contrast starts to decrease to less than 180°. When the temperature reaches 170 °C, the phase contrast becomes ambiguous, suggesting a transition from a T phase to a cubic (C) phase.

To summarize, PZN-7%PT undergoes an R → T phase transition at approximately 100 °C, and the T → C phase transition occurs between 140 °C and 170 °C. Based on the revised phase diagram of PZN-x%PT proposed by Chang et al., the de-poling temperature and the Curie temperature (*T*_C_) for PZN-7%PT are approximately 97 °C and 160 °C, respectively [18], which is in good agreement with our results. This indicates that investigating the phase transition process of PZN-x%PT single crystals by directly observing the domain structure evolution via the PFM technique is feasible and reliable.

## 4. Conclusions

The domain structure and polarization rotation path of a [001]-oriented PZN-7%PT single crystal were studied by measuring the x-LPFM, y-LPFM, and VPFM images with a rotation stage at various temperatures. It was found that the local domain structure was a rhombohedral (R) phase with nanoscale twins at room temperature. Then, the domain structure changed to a tetragonal (T) phase at approximately 100 °C through an adaptive monoclinic (Mc) phase. The domain structure remained tetragonal (T) from 100 °C to 120 °C and transformed from the T phase to the C phase at temperatures between 140 °C and 170 °C. Therefore, the phase transition sequence of the [001]-cut PZN-7%PT single crystal with increasing temperature was R → Mc → T → C, which provides direct visualization of the local domain structure during the phase transition of PZN-7%PT single crystals.

## Figures and Tables

**Figure 5:**
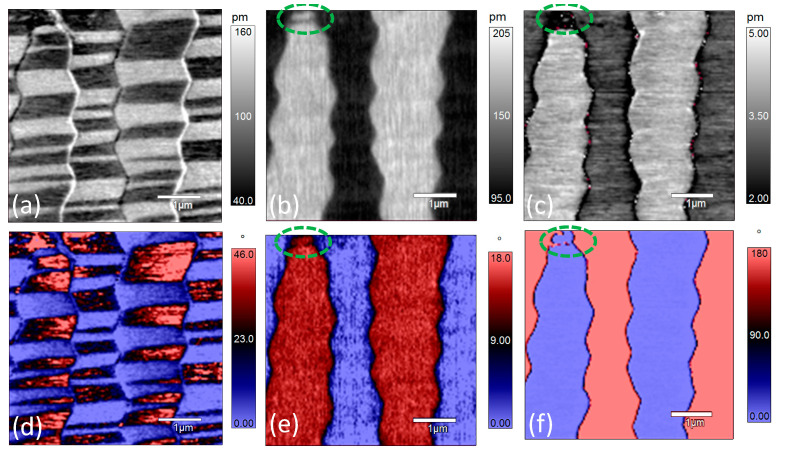
PFM amplitude and phase images of [001]-cut PZN-7%PT single crystal at 75 °C: (**a**–**c**) amplitude images of x-LPFM, y-LPFM, and VPFM, respectively; and (**d**–**f**) phase images of x-LPFM, y-LPFM, and VPFM, respectively.

## Data Availability

Data sharing is not applicable to this article.

## Supplementary Materials

The following are available online at https://www.mdpi.com/1996-1944/14/4/855/s1, Figure S1: PFM amplitude and phase of [001]-cut PZN-7%PT single crystal at 100 °C: (a–c) amplitude images of x-LPFM, y-LPFM and VPFM, respectively; and (d–f) phase images of x-LPFM, y-LPFM and VPFM, respectively, Figure S2: PFM amplitude and phase of [001]-cut PZN-7%PT single crystal at 140 °C: (a–c) amplitude images of x-LPFM, y-LPFM and VPFM, respectively; and (d–f) phase images of x-LPFM, y-LPFM and VPFM, respectively, Figure S3: PFM amplitude and phase of [001]-cut PZN-7%PT single crystal at 150 °C: (a–c) amplitude images of x-LPFM, y-LPFM and VPFM, respectively; and (d–f) phase images of x-LPFM, y-LPFM and VPFM, respectively.
