# Supplementary material for: Temperature-Induced Phase Transition Characteristics of [001]-Oriented 0.93Pb(Zn1/3Nb2/3)O3-0.07PbTiO3 (PZN-7%PT) Single Crystal by Using Piezoresponse Force Microscopy
Source: Materials (Basel). 2021 Feb 10;14(4):855. doi: 10.3390/ma14040855 (PMC7916775; doi:10.3390/ma14040855)
Supplement: Supplementary file 1 [file materials-14-00855-s001.zip › materials-1091048-supplementary.pdf]

materials

MDPI

Supplementary Materials

Temperature-Dependent Phase Transition Characteristics of [001]-Oriented $Pb(Zn_{1/3}Nb_{2/3})O_3$ – 7%$PbTiO_3$ (PZN – 7%PT) Single Crystal by Using Piezoresponse Force Microscopy

Wang Hongli and Kaiyang Zeng

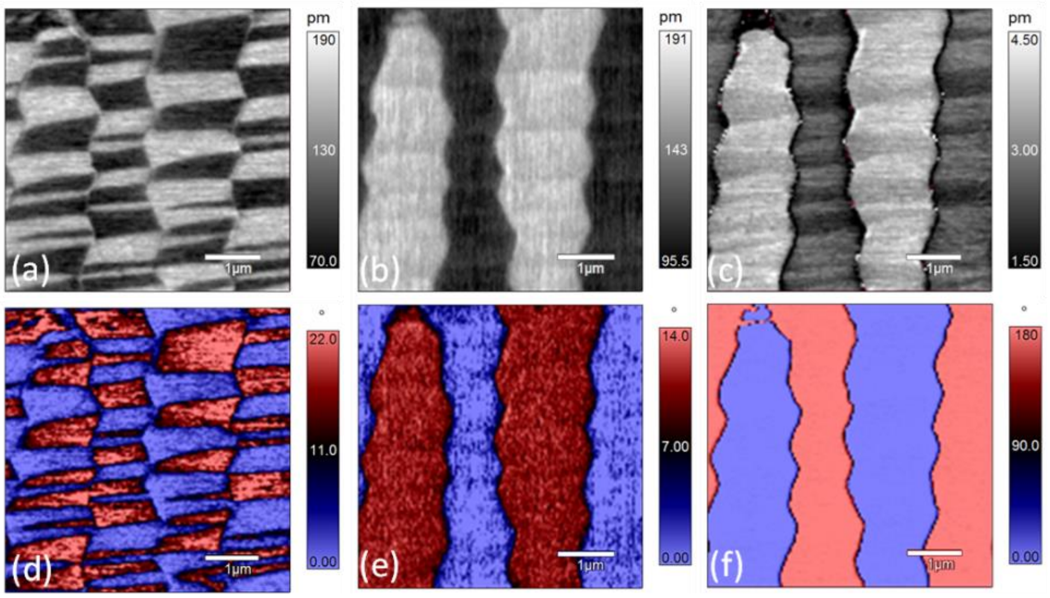

Figure S1. PFM amplitude and phase of (001)-cut PZN-7%PT single crystal at 100°C: (a)-(c) amplitude images of x-LPFM, y-LPFM and VPFM, respectively; and (d)-(f) phase images of x-LPFM, y-LPFM and VPFM, respectively.

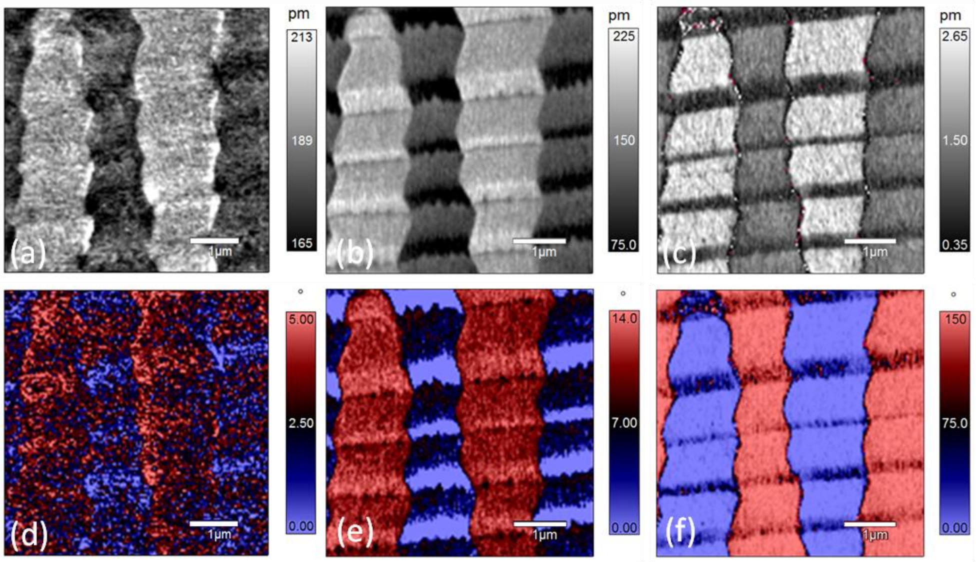

Figure S2. PFM amplitude and phase of (001)-cut PZN-7%PT single crystal at 140°C: (a)-(c) amplitude images of x-LPFM, y-LPFM and VPFM, respectively; and (d)-(f) phase images of x-LPFM, y-LPFM and VPFM, respectively.

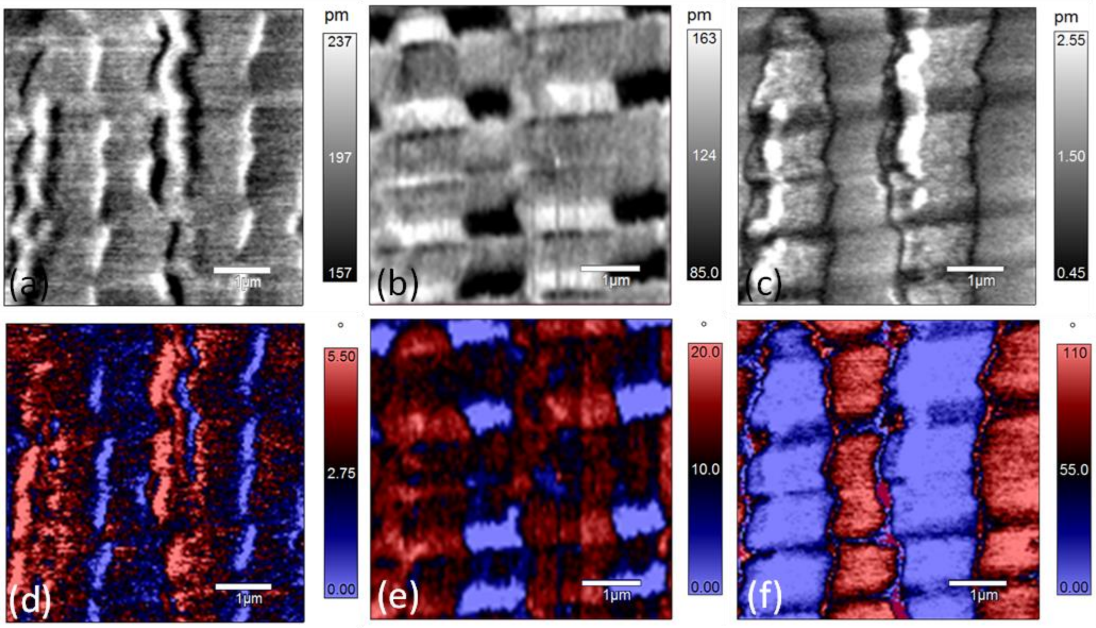

Figure S3. PFM amplitude and phase of (001)-cut PZN-7%PT single crystal at 150°C: (a)-(c) amplitude images of x-LPFM, y-LPFM and VPFM, respectively; and (d)-(f) phase images of x-LPFM, y-LPFM and VPFM, respectively.
